# Symbolic dimensions of health economics: connections with nursing work in hospitals

**DOI:** 10.1590/0034-7167-2023-0470

**Published:** 2025-09-01

**Authors:** Adriana do Prado Rodrigues Carneiro, Zenith Rosa Silvino, Sabrina da Costa Machado Duarte, Thiago Privado da Silva, Laura Johanson da Silva, Ítalo Rodolfo Silva

**Affiliations:** I Universidade Federal do Rio de Janeiro. Rio de Janeiro, Rio de Janeiro, Brazil; II Universidade Federal Fluminense. Niterói, Rio de Janeiro, Brazil; III Universidade Federal do Estado do Rio de Janeiro. Rio de Janeiro, Rio de Janeiro, Brazil

**Keywords:** Health Care Economics and Organizations, Nursing, Hospitals, Nursing Care, Hospital Administration., Economía y Organizaciones para la Atención de la Salud, Enfermería, Hospitales, Atención de Enfermería, Administración Hospitalaria.

## Abstract

**Objectives:**

to understand, from nurses’ perspective, the intervening conditions related to the meanings of health economics and nursing work in hospital settings.

**Methods:**

qualitative research, conducted in a university hospital, with 18 nurses, whose theoretical and methodological frameworks were, respectively, Symbolic Interactionism and Grounded Theory.

**Results:**

in the context of micropolitics, nurses understand the intervening conditions related to the connections between health economics and the work they perform. However, they also attribute meanings directed at macropolitics when they recognize health economics as a political element that is governed by decision-makers distant from nursing, but that directly affect the management of material and human resources in health.

**Final Considerations:**

nurses recognize themselves as a strategic workforce to drive health economics, in hospital settings, based on the positions they assume in these scenarios, mainly due to the nature of the work they perform in hospitals.

## RESUMEN

**Objetivos:**

comprender, desde la perspectiva del enfermero, las condiciones intervinientes relacionadas con los significados de la economía de la salud y el trabajo de enfermería en el contexto hospitalario.

**Métodos:**

investigación cualitativa, realizada en un hospital universitario, con 18 enfermeros, cuyos referentes teóricos y metodológicos fueron, respectivamente, el Interaccionismo Simbólico y la Teoría Fundamentada.

**Resultados:**

en el ámbito de la micropolítica, los enfermeros comprenden las condiciones intervinientes relacionadas con las conexiones entre la economía de la salud y el trabajo que desempeñan. Sin embargo, también atribuyen significados dirigidos a la macropolítica cuando reconocen la economía de la salud como un elemento político que es gobernado por tomadores de decisiones alejados de la enfermería, pero que incide directamente en la gestión de los recursos materiales y humanos en salud.

**Consideraciones Finales:**

los enfermeros se reconocen como una fuerza laboral estratégica para dinamizar la economía de la salud, en el contexto hospitalario, a partir de las posiciones que asumen en estos escenarios, principalmente por la naturaleza del trabajo que desempeñan en el hospital.

## INTRODUCTION

Scientific progress in health economics (HE) began to configure, in a global scenario, an emerging need for scientific responses from the 1960s onwards, a context in which universities intensified studies on economic assessment in health, mainly with the participation of healthcare professionals in dialogues related to the topic^(1)^. In this context, HE began to form a discipline that brings together economic and health knowledge, with the objective of sustainable use of material and human resources^(2)^.

Initially, with the aim of fostering the production of knowledge about economics among healthcare professionals, as well as assisting them in the decision-making process to, among other consequences, favor a return on investments in the health sector^(3)^, HE is currently expanding paradigmatic possibilities, especially when it aims to achieve better conditions for the sustainability implied in the health sector. Sustainability, in this context, is understood as the rational use of material resources based on awareness capable of understanding the importance of rational consumption of finite goods focused on current and future needs that are anticipated for generations to come^(4)^.

In the case of the health sector, it is important to highlight it as a multifaceted context that therefore involves different cultures, principles and guidelines that can affect the work process of the professionals involved^(5)^. This understanding implies that the contextual specificities of health settings can also influence the field of meanings and actions aimed at HE.

Thus, hospital settings assume significant importance for discussions related to HE, especially because these settings involve high technological density, which generates the need for high investments^(4)^, as well as significant consumption of resources and disposal of solid health waste^(6)^, for instance. Furthermore, public investments in hospitals consume a considerable portion of the health sector’s budget.

Regarding the complexity involved in HE, it is clear that this is made up of a set of interactions, systematically supported by scientific knowledge, technical skills, relational, behavioral and attitudinal skills of healthcare professionals and the like^(1)^. In this reality, nursing assumes relevant importance, not only due to the number of professionals involved in caring for hospitalized patients, but mainly due to the nature of care practices developed by these professionals, uninterruptedly^(7,8)^. However, in contrast to this reality, there is the symbolic dimension, developed and maintained by decision-makers in central areas of management of different health sectors who, mistakenly, attribute negative implications to nursing related to spending on resources destined for health^(9)^, when evidence points to the opposite, by signaling the importance of investments in nursing as conditions to positively impact the economy and healthcare systems of nations^(8,10-12)^.

Global agendas, as supported by the United Nations (UN) to achieve the Sustainable Development Goals of the 2030 Agenda, reinforce the importance of nursing for higher education based on a sustainable perspective that directly and indirectly affects the conscious consumption of resources involved in providing healthcare to people, as well as the role of nursing in the development of the economy through the strengthening of nations’ healthcare systems^(12,13)^.

In a decentralized process, HE also affects the quality of life of patients and their families, when it promotes the ability to reduce costs to patients with technological resources, such as the rational use of medications during hospitalization/hospital discharge processes as well as the ability of nursing to establish quality of care that allows for a reduction in length of hospital stay^(8)^. However, it is necessary for nurses themselves to recognize their importance for HE; to do so, it is necessary to know the conditions that influence the way nurses perceive this reality, especially based on the influence they can exert on it.

To understand the above problem, we take Symbolic Interactionism (SI) as an epistemological basis, whose premises support the understanding that individuals interpret facts and act on them according to the meanings they attribute to the reality with which they interact, and that these meanings are the result of social interaction processes, which change over time^(14)^.

Therefore, it is important for health and for nursing itself to understand the meanings of HE attributed by professionals who make up the largest group of human resources in the health sector and who, in addition to their numerical expression, can take on a strategic position capable of enabling improvements in HE based on sustainable practices in work processes in the health sector. Therefore, it is worth asking: what conditions (intervening/contextual) influence the meanings of HE revealed by nurses in hospital settings?

## OBJECTIVES

To understand, from nurses’ perspective, the intervening conditions related to the meanings of HE and nursing work in hospital settings.

## METHODS

### Ethical aspects

This research was approved in August 2021 by the Research Ethics Committee. It met the requirements of Resolutions 466/12 and 580/18 of the Brazilian National Health Council. After consenting, participants signed the Informed Consent Form. To guarantee study participant anonymity, their identities were preserved using alphanumeric indications. Thus, throughout the article, they were identified using the letters “Nur” (Nur.), followed by the number of their respective interviews.

### Theoretical-methodological framework

The theoretical and methodological frameworks of this research were, respectively, Symbolic Interactionism^(14)^ and Grounded Theory (GT)^(15)^. Symbolic Interactionism assumes a perspective of interpretation of reality according to which it allows us to understand how an individual interprets their own reality and the objects inserted in it^(15)^. As for GT, it is a developed method that is based on a set of analytical resources that favor the understanding of factors that structure, condition and/or influence a given social phenomenon^(15,16)^. Thus, the methodological framework is aligned with the theoretical framework when it positions the analysis and interpretation of results based on a multidimensional, social and symbolic perspective, which considers the need for contextual, cultural, individual and social in-depth analysis to understand the phenomena investigated^(16,17)^.

### Study design

This is qualitative research, of the explanatory type, by correlating subcategories (principles) around a category (concept) to explain the reality investigated. For the research structure, Enhancing the QUAlity and Transparency of Health Research (EQUATOR) guidelines were used notably the COnsolidated criteria for REporting Qualitative research (COREQ).

### Methodological procedures

The collected data were coded and were guided by the analytical resources of GT, the first of which was the open coding process. In this phase, the data that were separated into distinct parts were rigorously examined and compared in search of similarities and differences.

The coding process considered the analysis “line by line”, when the researchers examine the data in detail “sentence by sentence”, “incident by incident” (by incident we consider the specific nature of data based on qualities, attributes or intensities attributed by deponents, in their interviews). This analysis is also considered as microanalysis, and it results in the first codes, still quite descriptive: preliminary codes^(16,17)^.

In the second phase, codes were grouped in the form of an axiom. This stage is called axial coding, which begins with a comparison between the preliminary codes that take on a more conceptual character and are therefore less descriptive of the raw data - interviews. This process results in conceptual data.

Subsequently, conceptual codes were grouped to form categories and subcategories, which are denser concepts, capable of assuming a higher level of abstraction in relation to the set of codes that supported them. Then, the integrative coding process began, the third phase of the coding process, formed through the comparison and analysis of categories and subcategories, carried out continuously, aiming to deepen the development of categories, integrate them and refine them^(16,17)^.

### Study setting

Data were collected at a federal university hospital in the city of Rio de Janeiro, RJ, which offers several clinical specialties and has approximately 550 beds. The study involved inpatient sectors, such as medical clinic, surgical clinic and Intensive Care Unit (ICU).

The aforementioned units were chosen intentionally, without any conflicts of interest, but only based on the hypothesis that the settings can influence the meanings of HE based on work processes that relate to routines of consumption of materials related to direct patient care. Thus, as all research aims to be replicated, the delimitation of units in a plural context such as that of a hospital assumes coherence for future comparisons in relation to potential similar studies.

### Data source

Eighteen nurses with two or more years of experience in patient care in a hospital setting and working as a nurse in a hospital setting in a medical clinic, surgical clinic, or ICU participated in the study. Nurses on vacation/leave of absence or on leave of any kind during the data collection period were excluded.

### Data collection and organization

Data collection took place from July to November 2022, through semi-structured interviews, conducted in in-person meetings, and took place individually, at the participants’ workplace, in calm and quiet environments. Audio recording was used and, subsequently, the raw data were transcribed using the Microsoft Word^®^ 97-2003 tool.

For data collection, semi-structured interviews were conducted, with the following leading questions: what does HE mean to you? How do you perceive yourself in this reality? What are the relationships between nursing and HE specifically in hospital settings? Based on the responses, circular questions were developed to facilitate the substantiated data gathering. The interviews took place in individual meetings, in a private environment, in the study setting itself, at previously agreed times that did not compromise the interviewees’ work activities.

The interviews lasted an average of 30 minutes each. The interviews were not repeated with the same participant. Analytical and reflective memos were used throughout the collection and analysis process, which occurred simultaneously in GT.

The memos allowed reflections on the development of concepts, thus helping to understand the theoretical data saturation, i.e., when the concept presents theoretical density, supported by its respective subcategories/principles. Furthermore, the process of theoretical saturation was discussed among the researchers before data collection was completed.

It is important to highlight that data collection was carried out by a nurse researcher with expertise in the data collection method and the adopted methodological framework. There was no conflict of interest related to professionals or the research setting.

### Data analysis

Data analysis was performed through the coding process, which followed the Straussian school principles, from the perspective of Corbin and Strauss^(16)^, namely: open, axial and integrative coding. The codes emerged from the coding process, which, when compared with each other and then grouped by conceptual similarity and difference, gave rise to categories and subcategories. The analysis tool known as the “paradigmatic model” was used to develop categories and subcategories. The method helps to answer fundamental questions about the phenomenon under study as well as to present it in a schematic form. In the research on screen, it was represented in its second version, highlighting the conditions component, which comprises the connections between causal, contextual and intervening conditions in the development of the phenomenon investigated^(16,17)^.

## RESULTS

Eighteen nurses participated in the study, whose characterization allowed the description of the following aspects: mean age of 39 years; mean time since training of ten years; mean time of professional experience of nine years. As for professional qualifications, (9) 56% were specialists; (6) 38% had master’s degree; (1) 6% had professional development. Concerning gender and work, (14) 78% of participants were female; (4) 22% were male; (7) 39% worked in the medical clinic; (6) 33% worked in the surgical clinic; and (5) 28% worked in the ICU.

Two categories were constructed, namely: 1) Symbolic dimensions of nursing on health economics and nursing work in the care of hospitalized patients; 2) Strengths and weaknesses in the connections between health economics and hospital nursing work. Each category is strengthened by the support of subcategories that attribute explanatory-informative abilities to them. Thus, the first category is presented.

### Symbolic dimensions of nursing on health economics and nursing work in the care of hospitalized patients

This category, supported by three subcategories, exposes the symbolic dimensions of nursing on HE and the several roles that nursing plays in the care of hospitalized patients, from administrative actions related to care management to direct patient care. Thus, in the subcategory “Health economics from nurses’ perspective”, participants considered that HE is related to: reducing material resource consumption; controlling these resources; the relationships between the reduction in material resource consumption and patient care quality, mediated by adequate nursing planning; the work processes of the health sector; and also the provision of human resources so that healthcare is provided adequately.

*The health economy is about reducing costs. Qualified care will also be related to reducing costs, because it will not exceed consumption.* (Nur. 1)

*It is prevention of recurrent hospitalizations, prevention of expenses with materials.* (Nur. 7)

*It is the entire process of reducing expenses linked to the health context.* (Nur. 8)

As a relational phenomenon, HE was also understood from a perception of the situation, which considers the importance of the context for its dynamics and functionality.

*I understand health economics as the entire functioning of the hospital, not only to manage its current resources, but also to predict future problems that have to do with the institution.* (Nur. 10)

*I understand it as something related to finances within the health sector, these being hospitals, Basic Health Units, services in which health is provided to the population.* (Nur. 14)

In addition to reducing consumption and controlling material, patrimonial, technological and human resources, HE was characterized by nurses as an integral part of the system that designs and organizes health itself as a service provision. Thus, HE, for the nurses investigated, is a political element that governs and manages health resources, in order to assist the population based on the current healthcare system.

*I think it is the system that designs and organizes the* [own] *healthcare system.* (Nur. 4)

*I think it is related to a body of the Ministry of Health that will distribute funds and implement health measures necessary for each population’s reality.* (Nur. 12)

In this way, HE was understood as a condition for the provision of resources capable of making healthcare viable.

[...] *is related to providing employees, materials, and procedures*. (Nur. 5)

*I understand it as being all expenses related to healthcare - what we use to care for patients, to carry out treatment, diagnosis, therapies.* (Nur. 11)

Since nursing is the largest professional category in the hospital sector, which, among other issues, consumes resources for the care it provides to patients, the subcategory “Perceptions of hospital nursing as a driving force influencing the health economy” highlights the profession as a significant influence when thinking about HE in the micro context of work and also in the macro context of the healthcare system. From this perspective, nurses see themselves as a driving force influencing HE due to the reduction in health costs that they lead, through the rational use of material resources.

[...] *there are several areas that are related, for instance, to reducing costs, not using excess materials.* (Nur. 1)

[...] *have control over the use of materials.* (Nur. 1)

[...] *I choose the materials to perform a dressing. When I use the resources in question with strategic thinking and objectives, I can have this perception of the impact of my work on the health economy.* (Nur. 3)

Care planning, understood as an administrative activity included in nursing care management, proved to be an important aspect in building the driving force behind nursing’s influence in higher education.

*Care planning, which will be carried out by me and the team, is made up of actions and strategies so that care is qualified and costs are reduced.* (Nur. 1)

*When a nurse performs the Systematization of Nursing Care efficiently, when they identify possible clinical deteriorations that may imply a longer hospital stay.* (Nur. 12)

In addition to the implications for nursing arising from the number of these professionals in relation to the other members of the multidisciplinary health team in the hospital, the research participants highlighted the dynamics of nursing professionals’ work based on the uninterrupted care they provide to hospitalized patients. According to the data, this situation results in a significant influence and specificity of nursing in HE.

*I believe that nursing can attend to patients’ needs much better than other members of the health team* [...] *we are caring for patients for 24 hours.* (Nur. 2)

[...] *for having all the hospital’s material resources at hand, for being in direct contact and for longer periods of time with patients and family members.* (Nur. 3)

The interactions between the nursing work process, which involves administrative and assistance dimensions related to the management of care for hospitalized patients, were, therefore, perceived as conditions that significantly affect HE, especially when this reality is compared to other healthcare professions by study participants.

*The work of a nurse in a hospital is broad, and we are responsible for many functions, such as managing the sector, managing beds, managing patient care, which involves the care plan, checking the discharge plan of the entire multidisciplinary team, requesting equipment/technology that patients need and that we do not have available in the sector, and requesting special medications. We are a reference in care. We have all the information about the patient that the multidisciplinary team needs.* (Nur. 16)

Since it is the dimension of meanings about a given object, the context can influence the symbolic interactions of this relational process. In this logic, there are, for instance, the conditions that interfere or modify professionals’ decision-making, such as availability of human resources, technological resources and materials for the care to be carried out. Furthermore, there are also reflections of the attitudes of organizational culture supported by institutional management and fed back by other sectors of the hospital. For study participants, this reality influences and is influenced by the model of distribution of material, technological and human resources of the institution itself, in addition to preferences for some sectors over others when it comes to management support for actions that affect HE.

*The way the hospital offers me, as a care manager, adequate human resources, in the correct number of professionals for each patient, this will impact the quality of care offered and also cost reduction.* (Nur. 1)

*In fact, we are linked to hospital management as a whole* [...] *it goes from management, purchasing services, warehouse, distribution, delivery, all of this to reach the assistance, to impact patients.* (Nur. 7*)*

[...] *it is a chain effect, a chain of work, a sequence. If there are failures in the processes, the negative effects are great.* (Nur. 7)

The subcategory “Perceived interactions between health economics and hospital nursing” demonstrates, based on the meanings revealed by participants, the impact of HE on nursing work in the hospital. This reality reveals nurses’ perspectives on different factors involved in the relationship between the management-care-economy process involved in the nursing work process based on the provision of human, material, technological and structural resources as factors that influence nursing in the hospital. Thus, for participants, nursing work cannot be carried out satisfactorily without due attention to HE that directly influences it.

[...] *in relation to being impacted by the economy, I believe that if the healthcare system works, we will have more complete teams, we will have an ideal dimensioning, we will have the necessary materials and technologies - always improving the quality of care.* (Nur. 4)

*When we have tools available to work, financial structure, our time will be optimized; a better line of care will be designed.* (Nur. 9)

*Yes, for instance, if the hospital does not have the appropriate material resources to perform that care, this impacts my care.* (Nur. 11)

In addition to the provision of the above-mentioned resources, the data highlighted the importance of paying due attention to the quality of these resources.

*Sometimes, I think it has a big impact. It depends on the quality of the material that is offered to us. How many times when performing a procedure have I had to open new materials because there is a non-conformity in the material you are using? It has a big impact! The economy predicts the best price, but the best price does not always have the best quality.* (Nur. 9)

In addition to providing sufficient and high-quality material resources for nurses, HE aims to ensure better working conditions for nursing professionals and professionals in the entire health sector. This reality seems to influence the construction of meanings and, consequently, nurses’ work in the hospital.

*So, the health economy influences nursing work in providing resources and working conditions; this influences my work. I will be more dedicated. I will not need to have another job, I will not work tired, I will have a single job. Consequently, with this, I will be in a better position to provide patients with a higher quality service. From the moment I offer a quality service, this patient of mine will return less, or seek less care in other services as a result of the good quality service they received.* (Nur. 18)

### Strengths and weaknesses in the connections between health economics and hospital nursing work

While the previous category emphasized the importance of connections between HE and nursing work in hospital settings, this category, in turn, revealed conditions that facilitate and limit connections between HE and nursing, even in hospital settings. Therefore, when understanding that nursing work, which results in care for hospitalized patients, occupies a space for articulation and negotiation in favor of achieving qualified care, the data indicated that there are not always adequate conditions for the nursing work process in that context. Thus, it is necessary to envision measures that reverse this reality; this is what was highlighted in the subcategory “Conditions that facilitate connections between health economics and nursing care for hospitalized patients”.

In this regard, nurses considered the following as facilitating measures for the connections between HE and nursing work in the care of hospitalized patients: knowledge of nursing professionals about HE and, consequently, training provided by the hospital related to HE; motivation and professional appreciation inclined towards actions that promote care designed from a cost-effectiveness perspective; flows that direct professional attitudes towards sustainable care, designed in quality and rational use of material resources, in addition to cost awareness for the institution, the health sector, the patient and the Brazilian State; quality of material resources made available; institutional and governmental attitudes in favor of appreciation of nursing professionals based on the understanding of the impact they have on the health sector and the economy; performance of leaders of bodies/entities linked to the profession, with emphasis on the Federal Nursing Council (In Portuguese, *Conselho Federal de Enfermagem* - COFEN).

[...] *training on the topic, flows. This will lead the professional to think about savings when managing care. It will also help us to have qualified assistance and reduce costs.* (Nur. 1)

*A motivated team greatly facilitates the interaction between care and the economy; the influence is great.* (Nur. 2)

[...] *the ideal amount of time I have available during my shift to be able to guide my patient will have a positive influence.* (Nur. 5).

*Professionals’ knowledge for the execution of their professional practice is, without a doubt, a condition that positively influences.* (Nur. 11)

*Professionals’ knowledge on the subject.* (Nur. 13; Nurse 18)

*The quality of materials that are offered to us to act on facilitates this interaction* [...] *attitudes of the rulers or bodies that govern us as well.* (Nur. 14)

*I think that something that facilitates this process of efficiency and effectiveness in nursing work is closely linked to professional appreciation* [...] *a valued professional works better.* (Nur. 18)

From another perspective, the subcategory “Limiting conditions of the interactive process between health economics and nursing care for hospitalized patients” revealed that there are conditions that limit/hinder the interaction between these two poles.

In relation to limiting conditions, the results indicated institutional situations that do not generate motivation for nursing professionals. For research participants, motivation builds professional attitudes that have an impact on higher education. In the meantime, the importance of interpersonal relationships at work was also highlighted, which, among other factors, nurses mention, are influenced by the allocation of funds to health institutions; in this case, in hospital settings.

For nurses, work relationships can be affected by the lack/scarcity of materials needed to provide quality care. In addition, this scarcity leads to team overload and job dissatisfaction.

[...] *we need to talk about motivation. If we are in a place where there is no good relationship, it generates physical and/or emotional exhaustion, and greatly impacts the relationship between care management and health economics.* (Nur. 2)

*I think it is a culture that needs to be changed in public hospitals, that of having reduced reserves. This negatively influences the relationship between the economy and the care we offer to patients.* (Nur. 7)

[...] *when there is a lack of materials, it becomes very difficult.* (Nur. 14)

[...] *the overload, the dissatisfaction of nursing teams, all of this influences our performance and what we will do. And those who need to double up will not act in a dissatisfied manner, because people have other commitments, besides working here.* (Nur. 15)

In addition to the aforementioned situations, there is an aggravating factor that presents itself as a barrier to the interaction between HE and nursing work in the hospital. This factor is related to the fragility of nursing knowledge about HE and also to the supposed lack of knowledge of the healthcare system regarding the connections between nursing and HE.

[...] *I can certainly say that nursing, in general, does not know about health economics. And they do not know because they have not been led to think about it. The healthcare system structure does not see this relationship between care management and health economics, so we will not see it.* (Nur. 14)

## DISCUSSION

The symbolic dimension of nurses regarding HE is supported by the understanding that, for its functionality, there is interdependence between the microeconomic/microcultural and macroeconomic/macrocultural spheres^(1,18)^, inserted from the conditions that directly affect patient care to the broader dimensions and governmental situations, for instance. Thus, nursing work is not distant from the growing concern with the economic factors that condition both the provision of healthcare services and the population’s own health indicators^(8,19)^. Both dimensions feed back into each other.

In line with the results of this study, especially when, for nurses, HE presents itself in a multifaceted way, from the reduction of the consumption of material resources in health to a sustainable practice, there is evidence that spending on hospitalization and treatment controls spending on health consumption with increasing repercussions, due to the high number of chronic diseases, the population aging process and the high technological density existing in hospital institutions^(20)^.

From another perspective, the construction of dialogues in organizational spaces for the conscious consumption of resources, in order to avoid waste and control costs, understood by the participants of this study as an important reality, establishes links with the understanding that the hospital is also a context conducive to human interactions affecting the process of knowledge and construction of values that direct sustainable praxis in the care of people^(20,21)^. Thus, it is clear that the actions developed in favor of economic management may be rooted in the meanings that professionals have constructed and value regarding the connections between what they perform and the impact they have on the economy^(18,21)^.

Furthermore, as nurses recognize themselves as a driving force for the establishment and maintenance of work processes capable of promoting sustainability in the health sector, through the rational use of material resources, for instance, the data support indications of a critical view of the profession in relation to its own importance for HE based on the numerical nature^(10,22)^, as well as the ontological nature of nursing know-how in care practices that involve the interdependence of managerial and care actions typical of nursing care management^(8,9)^. Thus, the research revealed that nursing care management is a link, in a microcultural context, in the approximation of macroeconomic and microeconomic policies in hospital settings for the inseparability of economy and health based on nursing work.

Among the premises of Symbolic Interactionism is the understanding that the meanings about a given object are socially constructed from interactions between people, but such interactions occur in contextual perspectives where the signified object emerges and develops^(14)^. In this logic, when dealing with the meanings that nurses reveal about HE and its interfaces with nursing work, it is necessary to support the idea that the development of economic culture in hospital institutions is fundamental. Actions such as organizational culture, training process of professionals, even within the scope of continuing education, flows and routines that favor the tendencies of established or prospective organizational culture, as well as actions that favor feedback on the meanings of professionals about the connections between their work and HE can be inferred from this process^(9,22,23)^.

Despite the above, it is not uncommon for there to be inconsistencies that interfere with the management of care based on negative implications arising from inefficient economic management and that also modify the field of meanings regarding the connections between these two dimensions, in such a way as to affect the work process itself when the weaknesses of HE result, for instance, in difficulties for professionals to make decisions based on the best evidence^(9,23-26)^, especially when there is a shortage of technological resources and qualified human resources.

The conditions that can combat the obstacles to connections between HE and hospital nursing work take into account the dynamics of the organizational context itself as a reality capable of reordering meanings already established in the cultural course of a given reality, when, through intentional action, it projects conditions for new perspectives on the same reality. From this situation, possibilities for a new interpretative process of subjects in the face of new meanings for the same object or situation are inferred^(14,26)^. In this research, actions such as training promoted by the hospital related to HE and flows that direct professional attitudes towards sustainable and quality care seem to support symbolic possibilities to project and/or strengthen meanings about HE and nursing.

It is therefore worth highlighting that the epistemological position of Symbolic Interactionism rejects the idea that the social world can be represented in terms of deterministic relationships, and moves in favor of a view that knowledge, understanding and explanations of social relationships take into account the social order, and are elaborated by human beings, in the way they are meaningful to them^(14)^. Thus, positioning oneself in the construction of strategies of motivation and professional appreciation inclined towards actions that promote care designed from a cost-effectiveness perspective is a condition capable of boosting the management of a health economy in an efficient way. Furthermore, from a macroeconomic perspective, there are governmental attitudes^(27,28)^, as well as attitudes of agencies/entities linked to the profession, with emphasis on COFEN. For this reason, it is admitted that studies on the topic at hand are necessary, in order to provide decision-makers with important information on the effective use of available resources to enhance the benefits of health promotion^(27)^, considering the contextual and symbolic reality of those involved in healthcare, in the different contexts where it is developed.

### Study limitations

Although the research presents the meanings of professionals who, under deontological force, have the role of coordinating the nursing team, the meanings revealed by the other nursing team members could add value to the data, in order to signal or not other factors that condition the connections between HE and nursing. This understanding assumes contextual/national implications, as it considers the Brazilian reality, in which nursing is composed of nurses, nursing technicians and assistants. Added to the above is the fact that the other nursing team members consume, in significant projection, material resources to perform nursing care. The reality of private hospitals, due to the organizational culture, may signal different data in relation to what was presented in the study in question.

### Contributions to nursing, health or public policy

The meanings revealed by nurses about HE, based on the hospital reality, provide possibilities for us to understand how these professionals perceive themselves in the face of the work they perform and which is affected by HE. At the same time, they demonstrate how nursing impacts, albeit indirectly, HE based on the ontological and numerical nature that they present in hospital institutions in patient care.

The symbolic interactions between HE and hospital nursing signal, based on the meanings revealed by nurses, the conditions that influence nursing and HE, from organizational flows, knowledge processes of these professionals for the sustainability of care and health, to institutional and governmental valorization measures capable of contemplating nursing based on the importance they have for HE.

## FINAL CONSIDERATIONS

It is within the scope of the micropolitics of work that nurses initially understand the intervening conditions related to the connections between HE and the work they perform in the hospital. However, these professionals also attribute meanings directed by macropolitics when they recognize HE as a political element that is governed by decision-makers who do not always meet nursing demands, but who directly affect the management of material and human resources in health, notably in hospital settings.

Nurses recognize themselves as a strategic workforce to promote higher education in hospital settings, based on the positions they take in these settings, whether due to their uninterrupted stays or the number of professionals, but mainly due to the nature of the work they perform based on the consumption and management of resources for the care of hospitalized patients. Furthermore, among the intervening conditions affecting the meanings of nurses about higher education and nursing work, there are actions that can be strategic, such as continuing education related to higher education, incentives and motivation of workers to better manage resources, leadership and policies that value nursing.
