# Supplementary material for: Symbolic dimensions of health economics: connections with nursing work in hospitals
Source: Rev Bras Enferm. 2025 Sep 1;78(Suppl 1):e20230470. doi: 10.1590/0034-7167-2023-0470 (PMC12407854; doi:10.1590/0034-7167-2023-0470)
Supplement: Translation

# Translation: Dimensões simbólicas sobre economia da saúde: conexões com o trabalho da enfermagem em hospitais

**DOI:** 10.1590/0034-7167-2023-0470pt

**Authors:** Adriana do Prado Rodrigues Carneiro, Zenith Rosa Silvino, Sabrina da Costa Machado Duarte, Thiago Privado da Silva, Laura Johanson da Silva, Ítalo Rodolfo Silva

**Affiliations:** I Universidade Federal do Rio de Janeiro. Rio de Janeiro, Rio de Janeiro, Brasil; II Universidade Federal Fluminense. Niterói, Rio de Janeiro, Brasil; III Universidade Federal do Estado do Rio de Janeiro. Rio de Janeiro, Rio de Janeiro, Brasil

**Keywords:** Economia e Organizações de Saúde, Enfermagem, Hospitais, Cuidados de Enfermagem, Administração Hospitalar.

## Abstract

**Objetivos:**

compreender, sob a perspectiva de enfermeiros, as condições intervenientes relacionadas aos significados sobre economia da saúde e trabalho da enfermagem no contexto hospitalar.

**Métodos:**

pesquisa qualitativa, realizada em hospital universitário, com 18 enfermeiros, cujos referenciais teórico e metodológico foram, respectivamente, o Interacionismo Simbólico e a Teoria Fundamentada nos Dados.

**Resultados:**

no âmbito da micropolítica, os enfermeiros compreendem as condições intervenientes relacionadas às conexões entre economia da saúde e o trabalho que exercem. Todavia, também atribuem significados direcionados à macropolítica quando reconhecem a economia da saúde como elemento político que é regido por tomadores de decisão distanciados da enfermagem, mas que afetam diretamente o gerenciamento de recursos materiais e humanos da saúde.

**Considerações Finais:**

os enfermeiros se reconhecem como força de trabalho estratégica para impulsionar a economia da saúde, no contexto hospitalar, a partir das posições que assumem nesses cenários, principalmente pela natureza do trabalho que realizam no hospital.

## INTRODUÇÃO

O progresso científico sobre economia da saúde (ES) passou a configurar, em cenário global, necessidade emergente de respostas científicas a partir da década de 1960, contexto em que as universidades intensificaram estudos da avaliação econômica em saúde, principalmente com a participação de profissionais de saúde nos diálogos relacionados ao tema^(1)^. Nessa conjuntura, a ES passou a conformar disciplina que agrega conhecimentos econômicos e de saúde, com o objetivo do uso sustentável dos recursos materiais e humanos^(2)^.

Com a finalidade, inicialmente, de fomentar a produção do conhecimento sobre economia aos profissionais de saúde, bem como auxiliá-los no processo de tomada de decisão para, entre outras consequências, favorecer retorno aos investimentos do setor da saúde^(3)^, a ES, atualmente, expande possibilidades paradigmáticas, sobretudo quando vislumbra alcançar melhores condições para a sustentabilidade implicada no setor da saúde. Por sustentabilidade, nessa conjuntura, compreende-se o uso racional de recursos materiais fundamentado em consciência capaz de compreender a importância do consumo racional de bens finitos focado nas necessidades atuais e futuras que são antecipadas para as gerações que estão por vir^(4)^.

Tratando-se do setor da saúde, cabe destacá-lo como contexto multifacetado que envolve, portanto, culturas, princípios e diretrizes distintas que podem afetar o processo de trabalho dos profissionais nele inseridos^(5)^. Depreende-se desse entendimento a compreensão de que as especificidades contextuais dos cenários da saúde podem influenciar, também, o campo dos significados e ações voltadas para a ES.

Desse modo, o contexto hospitalar assume importância significativa para as discussões relacionadas à ES, especialmente porque esses cenários envolvem alta densidade tecnológica, que gera a necessidade de elevados investimentos^(4)^, bem como acentuado consumo de recursos e descarte de resíduos sólidos de saúde^(6)^, por exemplo. Ademais, os investimentos públicos com os hospitais consomem considerada parcela orçamentária do setor da saúde.

Tratando-se da complexidade envolvida na ES, percebe-se que esta é constituída pelo conjunto de interações, sistematicamente sustentadas em conhecimentos científicos, competências técnicas, habilidades relacionais, comportamentais e de atitudes dos profissionais de saúde e afins^(1)^. Nessa realidade, a enfermagem assume relevante importância, não somente pela quantidade de profissionais envolvidos no cuidado ao paciente hospitalizado, mas, principalmente, pela natureza das práticas de cuidado, desenvolvidas por esses profissionais, de forma ininterrupta^(7,8)^. Contudo, em contraste com essa realidade, tem-se a dimensão simbólica, desenvolvida e mantida por tomadores de decisão em âmbitos centrais da gestão de diferentes setores da saúde que, equivocadamente, imputam à enfermagem implicações negativas relacionadas aos gastos com os recursos destinados à saúde^(9)^, quando evidências direcionam o oposto, ao sinalizarem a importância de investimentos na enfermagem como condições para impactar positivamente a economia e os sistemas de saúde das nações^(8,10-12)^.

Agendas globais, tal qual sustentadas pela Organização das Nações Unidas (ONU) para o atingimento dos Objetivos de Desenvolvimento Sustentável da Agenda 2030, reforçam a importância da enfermagem para a ES pautada em uma perspectiva sustentável que afeta direta e indiretamente o consumo consciente de recursos implicados na prestação da assistência à saúde das pessoas, bem como o papel da enfermagem para o desenvolvimento da economia mediante o fortalecimento dos sistemas de saúde das nações^(12,13)^.

Em um processo descentralizado, a ES afeta também a qualidade de vida dos pacientes e de suas famílias, quando fomenta a capacidade de menores custos ao paciente com recursos tecnológicos, como uso racional de medicamentos no decorrer dos processos de hospitalização/alta hospitalar, bem como pela capacidade de a enfermagem estabelecer qualidade da assistência que permita a redução do tempo de internação^(8)^. Todavia, faz-se necessário que os próprios enfermeiros reconheçam a sua importância para a ES; para tanto, há que se conhecer as condições que influenciam a forma como os enfermeiros percebem essa realidade, especialmente a partir da influência que podem nela exercer.

Para a compreensão da problemática supra, tomamos como base epistemológica o Interacionismo Simbólico (IS), cujas premissas favorecem sustentação para o entendimento de que os indivíduos interpretam os fatos e agem perante eles de acordo com os significados que atribuem à realidade com a qual interagem, e que esses significados são resultado dos processos de interação social, que se modificam ao longo do tempo^(14)^.

Portanto, importa para a saúde e para a própria enfermagem compreender os significados sobre ES, atribuídos pelos profissionais que conformam o maior grupo de recursos humanos do setor da saúde e que, além de sua expressão numérica, podem assumir posição estratégica capaz de viabilizar melhoras na ES a partir de práticas sustentáveis nos processos de trabalho no setor da saúde. Sendo assim, cabe questionar: que condições (intervenientes/contextuais) influenciam os significados sobre ES desvelados por enfermeiros no contexto hospitalar?

## OBJETIVOS

Compreender, sob a perspectiva de enfermeiros, as condições intervenientes relacionadas aos significados sobre ES e trabalho da enfermagem no contexto hospitalar.

## MÉTODOS

### Aspectos éticos

Esta pesquisa foi aprovada em agosto de 2021 pelo Comitê de Ética e Pesquisa com Seres Humanos. Atendeu às exigências das Resoluções nº 466/12 e nº 580/18 do Conselho Nacional de Saúde. Após o consentimento, os participantes assinaram o Termo de Consentimento Livre e Esclarecido. Para garantir o anonimato dos participantes do estudo, suas identidades foram preservadas a partir de indicações alfanuméricas. Desse modo, ao longo do artigo, foram identicados usando as letras “Enf” (enfermeiro), seguidas do número das suas respectivas entrevistas.

### Referencial teórico-metodológico

Os referenciais teórico e metodológico da pesquisa foram, respectivamente, o Interacionismo Simbólico^(14)^ e a Teoria Fundamentada em Dados (TFD)^(15)^. O Interacionismo Simbólico assume uma perspectiva de interpretação da realidade segundo a qual permite compreender como o indivíduo interpreta a própria realidade e os objetos nela inseridos^(15)^. Sobre a TFD, trata-se de um método desenvolvido que tem como alicerce um conjunto de recursos analíticos que favorecem a compreensão sobre os fatores que estruturam, condicionam e/ou influenciam um dado fenômeno social^(15,16)^. Desse modo, o referencial metodológico está alinhado ao referencial teórico quando posiciona a análise e interpretação dos resultados com base em uma perspectiva multidimensional, social e simbólica, que considera a necessidade de aprofundamentos contextuais, culturais, individuais e sociais para a compreensão dos fenômenos investigados^(16,17)^.

### Tipo de estudo

Pesquisa qualitativa, do tipo explicativa, por correlacionar subcategorias (princípios) em torno de categoria (conceito) para explicar a realidade investigada. Para a estrutura da pesquisa, foram empregadas diretrizes do *Enhancing the QUAlity and Transparency Of health Research* (EQUATOR), notadamente o guia *Consolidated criteria for REporting Qualitative research* (COREQ).

### Procedimentos metodológicos

Os dados coletados foram codificados e tiveram como guia metodológico os recursos analíticos da TFD, sendo o primeiro deles o processo de codificação aberta. Nessa fase, os dados que foram separados em partes distintas foram rigorosamente examinados e comparados em busca de similaridades e diferenças.

O processo de codificação considerou a análise “linha por linha”, ocasião em que os pesquisadores examinam detalhadamente os dados “frase por frase”, “incidente por incidente” (por incidente considera-se a natureza específica dos dados a partir de qualidades, atributos ou intensidades atribuídas pelos depoentes, em suas entrevistas). Essa análise é também considerada como microanálise, e dela resultam os primeiros códigos, ainda bastante descritivos: os códigos preliminares^(16,17)^.

Na segunda fase, ocorreu o processo de agrupamento dos códigos em forma de axioma. Esta etapa é denominada de codificação axial, que é iniciada a partir da comparação entre os códigos preliminares que passam a assumir um caráter mais conceitual e, portanto, menos descritivos dos dados brutos - entrevistas. Desse processo, resultam os dados conceituais.

Adiante, ocorreu o agrupamento dos códigos conceituais para formar as categorias e subcategorias, que são conceitos mais densos, capazes de assumir maior nível de abstração em relação ao conjunto de códigos que os sustentaram. Em seguida, iniciou-se o processo de codificação integrativa, a terceira fase do processo de codificação, formada através da comparação e análise das categorias e subcategorias, realizado de forma contínua, objetivando aprofundar o desenvolvimento das categorias, integrá-las e refiná-las^(16,17)^.

### Cenário do estudo

Os dados foram coletados em hospital universitário federal na cidade do Rio de Janeiro, RJ, que apresenta diversas especialdiades clínicas, contendo cerca de 550 leitos. O estudo envolveu os setores de internação, como clínica médica, clínica cirúrgica e Unidade de Terapia Intensiva (UTI).

A escolha das unidades supracitadas ocorreu de forma intencional, sem quaisquer conflitos de interesse, mas apenas sob sustentação da hipótese de que os cenários podem influenciar os significados sobre ES com base nos processos de trabalhos que se relacionam com as rotinas de consumo de materiais afetas ao cuidado direto ao paciente. Desse modo, como toda pesquisa visa à sua replicação, a delimitação de unidades em um contexto plural como o de um hospital assume coerência para futuras comparações em relação aos potenciais estudos assemelhados.

### Fonte de dados

Participaram da pesquisa 18 enfermeiros com tempo de experiência no cuidado ao paciente no contexto hospitalar igual ou superior a dois anos e inseridos no processo de trabalho como enfermeiro no contexto hospitalar em unidade clínica médica, clínica cirúrgica, ou UTI. Enfermeiros de férias/afastamento ou de licença de qualquer modalidade durante o período da coleta de dados foram excluídos.

### Coleta e organização dos dados

A coleta de dados ocorreu no período de julho a novembro de 2022, por meio de entrevistas semiestruturadas, realizadas em encontros presenciais, e aconteceu de forma individualizada, em local laboral dos participantes, em ambientes calmos e silenciosos. Contou com recurso de gravação de áudio e, posteriormente, ocorreu a transcrição dos dados brutos através da ferramenta da *Microsoft Word* ^®^ 97-2003.

Para a coleta de dados, foram realizadas entrevistas semiestruturadas, cujas perguntas indutoras foram: o que significa ES para você? Como você se percebe nessa realidade? Para você, quais as relações entre enfermagem e ES especificamente no contexto hospitalar? A partir das respostas, perguntas circulares foram elaboradas, de modo a favorecer a apreensão consubstanciada dos dados. As entrevistas aconteceram em encontros individuais, em ambiente reservado, no próprio cenário de estudo, em horários previamente acordados e que não comprometessem as atividades laborais dos entrevistados.

As entrevistas tiveram duração média de 30 minutos cada. As entrevistas não foram repetidas com um mesmo participante. Foram empregados memorandos analíticos e reflexivos durante todo o processo de coleta e análise, processo este que ocorreu de forma simultânea na TFD.

Os memorandos permitiram reflexões sobre o desenvolvimento dos conceitos, de modo que auxiliaram na compreensão sobre a saturação teórica dos dados, isto é, quando o conceito apresenta densidade teórica, sustentado em suas respectivas subcategorias/princípios. Ademais, o processo de saturação teórica fora discutido entre os pesquisadores antes da coleta de dados ser finalizada.

Cumpre destacar que a coleta de dados ocorreu com pesquisadora enfermeira com *expertise* no método de coleta de dados e no referencial metodológico adotado. Não houve nenhum conflito de interesse relacionado aos profissionais ou ao cenário da pesquisa.

### Análise dos dados

A análise dos dados se deu por meio do processo de codificação, que seguiu os princípios da escola straussiana, da perspectiva de Corbin e Strauss^(16)^, a saber: codificação aberta, axial e integrativa. Do processo de codificação, surgiram os códigos, que, quando comparados entre si e, então, agrupados por similaridade e diferença conceitual, fizeram emergir as categorias e subcategorias. Para o desenvolvimento das categorias e subcategorias, utilizou-se a ferramenta de análise conhecida como “modelo paradigmático”. O método auxilia a responder questões fundamentais sobre o fenômeno de estudo, bem como apresentá-lo de forma esquematizada. Na pesquisa em tela, foi representado em sua segunda versão, destacando-se o componente condições, que compõe as conexões entre condições causais, contextuais e intervenientes ao desenvolvimento do fenômeno investigado^(16,17)^.

## RESULTADOS

Participaram da pesquisa 18 enfermeiros, cuja caracterização possibilitou a descrição das seguintes vertentes: média de idade de 39 anos; tempo médio de formação de dez anos; média de tempo de experiência profissional de nove anos. Em relação à qualificação profissional, (9) 56% eram especialistas; (6) 38% eram mestres; (1) 6% possuíam aperfeiçoamento profissional. Em relação ao sexo e trabalho, (14) 78% dos participantes eram do sexo feminino; (4) 22% eram do sexo masculino; (7) 39% trabalhavam na clínica média; (6) 33% trabalhavam na clínica cirúrgica; e (5) 28% trabalhavam na UTI.

Duas categorias foram construídas, nomeadamente: 1) Dimensões simbólicas da enfermagem sobre economia da saúde e trabalho da enfermagem no cuidado ao paciente hospitalizado; 2) Potencialidades e fragilidades nas conexões entre economia da saúde e trabalho da enfermagem hospitalar. Cada categoria se fortalece a partir do apoio de subcategorias que lhes atribuem habilidades explicativo-informativas. Desse modo, apresenta-se a primeira categoria.

### Dimensões simbólicas da enfermagem sobre economia da saúde e trabalho da enfermagem no cuidado ao paciente hospitalizado

Esta categoria, sustentada por três subcategorias, expõe as dimensões simbólicas da enfermagem sobre ES e os múltiplos papéis que a enfermagem desempenha no cuidado ao paciente hospitalizado, desde ações administrativas afetas ao gerenciamento do cuidado até assistência direta ao paciente. Desse modo, na subcategoria “Economia da saúde a partir do olhar do enfermeiro”, os participantes consideraram que a ES está relacionada: à redução do consumo dos recursos materiais; ao controle desses recursos; às relações entre a redução do consumo de recursos materiais e qualidade da assistência ao paciente, mediada por planejamento adequado da enfermagem; aos processos de trabalho do setor da saúde; e também à provisão de recursos humanos para que a assistência à saúde aconteça de forma adequada.

*A economia da saúde é a redução de custos. Uma assistência qualificada vai ter também relação com a diminuição dos custos, porque não vai extrapolar o consumo.* (Enf.1)

*prevenção de internações recorrentes, prevenção de gastos com materiais.* (Enf.7)

*É todo o processo de redução de gastos vinculado ao contexto de saúde.* (Enf.8)

Como fenômeno relacional, a ES foi compreendida, também, a partir de uma percepção de conjuntura, que considera a importância do contexto para a sua dinâmica e funcionalidade.

*Eu compreendo como economia da saúde toda a parte de funcionamento do hospital, não somente para gerir os recursos próprios atuais, mas também para prever futuros problemas que tenham a ver com a instituição.* (Enf.10)

*Compreendo como algo relacionado a finanças dentro do setor da saúde, sendo esses hospitais, Unidades Básicas de Saúde, serviços em que a saúde é prestada à população.* (Enf.14)

Além da redução do consumo e controle dos recursos materiais, patrimoniais, tecnológicos e humanos, a ES foi caracterizada pelos enfermeiros como parte integrante do sistema que desenha e organiza a própria saúde como prestação de serviço. Desse modo, a ES, para os enfermeiros investigados, é elemento político que rege e gerencia os recursos da saúde, de modo a atender à população a partir do sistema de saúde vigente.

*Eu penso que é o sistema que desenha e organiza o* [próprio] *sistema de saúde.* (Enf.4)

*Penso que está relacionada a um órgão do Ministério da Saúde que irá distribuir verbas e aplicar medidas de saúde necessárias para cada realidade da população.* (Enf.12)

Desse modo, a ES foi compreendida como condição para a provisão de recursos capaz de viabilizar a própria assistência à saúde.

[...] *está relacionada com o prover de funcionários, de materiais, de procedimentos.* (Enf.5)

*Eu entendo como sendo todos os gastos relativos ao cuidado em saúde - aquilo que usamos para cuidar do paciente, para fazer o tratamento, diagnóstico, terapias.* (Enf.11)

Por ser a enfermagem a maior categoria profissional no setor hospitalar que, entre outras questões, consome recursos para os cuidados que realiza junto ao paciente, a subcategoria “Percepções da enfermagem hospitalar como força motriz de influência na economia da saúde” apresenta a profissão em destaque como influência significativa ao pensar ES no micro contexto do trabalho e, também, no macro contexto do sistema de saúde. Nessa perspectiva, os enfermeiros se percebem como força motriz de influência na ES, em função da redução de custos na saúde que protagonizam, através do uso racional dos recursos materiais.

[...] *há várias áreas que estão relacionadas, por exemplo, à diminuição dos custos, não usar materiais em excesso*. (Enf.1)

[...] *ter controle no uso dos materiais.* (Enf.1)

[...] *escolho os materiais para realizar um curativo. Quando utilizo os recursos em questão com o pensamento e objetivo estratégico, consigo ter essa percepção de impacto do meu trabalho com a economia da saúde.* (Enf.3)

O planejamento do cuidado, entendido como atividade administrativa inserida no gerenciamento do cuidado de enfermagem, revelou-se como aspecto importante na construção da força motriz da influência da enfermagem na ES.

*O planejamento do cuidado, que será realizado por mim e pela equipe, é feito de ações, estratégias, para que a assistência seja qualificada e ocorra a redução de custos.* (Enf.1)

*Quando o enfermeiro realiza a Sistematização da Assistência de Enfermagem de forma eficiente, quando identifica possíveis deteriorações clínica que poderão implicar em maior tempo de internação.* (Enf.12)

Além das implicações da enfermagem decorrentes da quantidade desses profissionais em relação aos demais membros da equipe multiprofissional de saúde no hospital, os participantes da pesquisa destacaram a dinâmica do trabalho dos profissionais de enfermagem a partir da assistência ininterrupta que realizam ao paciente hospitalizado. Dessa conjuntura, resulta, segundo os dados, significativa influência e especificidade da enfermagem na ES.

*Acredito que a enfermagem consegue assistir muito melhor as necessidades dos pacientes do que os demais membros da equipe de saúde* [...] *estamos cuidando do paciente por 24 horas.* (Enf.2)

[...] *por ter em mãos todos os recursos materiais do hospital, por estar em contato direto e por mais tempo com os pacientes e familiares*. (Enf.3)

As interações entre o processo de trabalho da enfermagem, que envolve dimensões administrativas e assistenciais afetas ao gerenciamento do cuidado à pessoa hospitalizada, foram, portanto, percebidas como condições que atravessam a ES de forma expressiva, sobretudo quando essa realidade é, pelos participantes do estudo, comparada às demais profissões de saúde.

*O trabalho do enfermeiro no hospital é amplo, e cabe a nós muitas funções, como o gerenciamento do setor, gerenciamento de leitos, gerenciamento do cuidado ao paciente, que envolve o plano de cuidado, verificação do plano de alta de toda equipe multiprofissional, solicitação de equipamentos/tecnologias que o paciente precisa e não temos disponíveis no setor, solicitação de medicações especiais. Somos referência no cuidado. Todas as informações sobre o paciente em que a equipe multiprofissional necessita, nós temos.* (Enf.16)

Por se tratar da dimensão dos significados sobre um determinado objeto, o contexto pode influenciar as interações simbólicas desse processo relacional. Nessa lógica, estão, por exemplo, as condições que interferem ou modificam a tomada de decisão do profissional, tais como disponibilidade de recursos humanos, disponibilidade de recursos tecnológicos e materiais para que o cuidado seja executado. Ademais, há também reflexos das atitudes da cultura organizacional sustentada pela direção institucional e retroalimentada por outros setores do hospital. Para os participantes do estudo, essa realidade influencia e é influenciada pelo modelo de distribuição dos recursos materiais, tecnológicos e humanos da própria instituição, além de predileções sobre alguns setores em detrimento de outros quando se trata de apoio da gestão para as ações que afetam a ES.

*O jeito que o hospital oferece pra mim, enquanto gestora do cuidado, recursos humanos adequados, em uma quantidade correta de profissionais para cada paciente, isso vai impactar na qualidade da assistência oferecida e também na redução de custos*. (Enf.1)

*Na verdade, a gente está ligado ao gerenciamento hospitalar como um todo* [...] *vai desde a direção, serviço de compras, almoxarifado, distribuição, entrega, tudo isso pra chegar na assistência, para impactar no paciente.* (Enf.7)

[...] *é um efeito em cadeia, um trabalho em cadeia, uma sequência. Se tiver falhas nos processos, os efeitos negativos são grandes.* (Enf.7)

A subcategoria “Interações percebidas entre economia da saúde e enfermagem hospitalar” é quem demonstra, a partir dos significados desvelados pelos participantes, os reflexos da ES para o trabalho da enfermagem no hospital. Depreendem-se dessa realidade as perspectivas dos enfermeiros sobre os diferentes fatores implicados na relação do processo gerência-cuidado-economia envolvidos no processo de trabalho da enfermagem a partir da disposição de recursos humanos, materiais, tecnológicos e estruturais como fatores que influenciam a enfermagem no hospital. Assim, para os participantes, o trabalho da enfermagem não se concretiza, de forma satisfatória, sem a devida atenção para a ES que o influencia diretamente.

[...] *em relação a ser impactada pela economia, acredito que se o sistema de saúde funcionar, teremos equipes mais completas, a gente vai ter um dimensionamento ideal, vamos ter materiais e tecnologias necessárias - sempre melhorando a qualidade do atendimento.* (Enf.4)

*Quando temos ferramentas disponíveis para trabalhar, estrutura financeira, o tempo nosso será otimizado; uma melhor linha de cuidado será desenhada.* (Enf.9)

*Sim, por exemplo, se o hospital não tem o recurso material adequado para executar aquele cuidado, isso impacta na minha assistência.* (Enf.11)

Além da provisão dos recursos acima mencionados, os dados destacaram a importância da devida atenção para a qualidade desses recursos.

*Às vezes, acho que impacta muito. Dependendo da qualidade do material que nos é oferecido. Quantas vezes ao fazer um procedimento, tive que abrir novos materiais, por existir não conformidade no material que você está usando? Impacta muito! A economia prevê o melhor preço, mas nem sempre o melhor preço terá melhor qualidade.* (Enf.9)

Além da provisão de recursos materiais em quantidade e qualidade para os enfermeiros, a ES visa garantir melhores condições de trabalho aos profissionais de enfermagem e de todo o setor da saúde. Essa realidade parece influenciar a construção dos significados e, por conseguinte, o trabalho dos enfermeiros no hospital.

*Então, a economia da saúde influencia o trabalho da enfermagem no prover de recursos e condições de trabalho; isso influencia no meu trabalho. Terei um zelo maior. Não precisarei ter outro vínculo, não vou trabalhar cansado, terei um emprego único. Consequentemente, com isso, terei condições melhores de dar ao paciente um serviço com maior qualidade. A partir do momento que ofereço um serviço de qualidade, esse meu paciente, ele vai retornar menos, ou buscando menos atendimento em outros serviços por reflexo de um serviço de boa qualidade que recebeu.* (Enf.18)

### Potencialidades e fragilidades nas conexões entre economia da saúde e trabalho da enfermagem hospitalar

Enquanto a categoria antecedente principiou a importância das conexões entre ES e trabalho da enfermagem no contexto hospitalar, esta, por sua vez, revelou condições facilitadoras e limitadoras para as conexões entre ES e enfermagem, ainda que no contexto hospitalar. Sendo assim, ao compreender que o trabalho da enfermagem, que resulta no cuidado ao paciente hospitalizado, ocupa espaço de articulação e negociação em prol da consecução do cuidado qualificado, os dados sinalizaram que nem sempre há condições adequadas para o processo de trabalho da enfermagem naquela conjuntura. Desse modo, faz-se necessário vislumbrar medidas que revertam tal realidade; é o que destacou a subcategoria “Condições facilitadoras das conexões entre economia da saúde e assistência da enfermagem ao paciente hospitalizado”.

Nessa direção, os enfermeiros consideraram como medidas facilitadoras das conexões entre ES e trabalho da enfermagem na assistência ao paciente hospitalizado: conhecimento dos profissionais de enfermagem sobre ES e, por conseguinte, treinamentos promovidos pelo hospital relacionados à ES; motivação e valorização profissional inclinadas para ações que impulsionem cuidados pensados em perspectiva custo-efetividade; fluxos que direcionem as atitudes profissionais a um cuidado sustentável, pensado em qualidade e uso racional de recursos materiais, além de consciência de custos para a instituição, para o setor da saúde, para o paciente e para o Estado brasileiro; qualidade dos recursos materiais disponibilizados; atitudes institucionais e governamentais em prol da valorização dos profissionais de enfermagem a partir da compreensão sobre o impacto que exercem no setor da saúde e economia; desempenho de lideranças dos órgãos/entidades vinculados à profissão, com destaque para o Conselho Federal de Enfermagem (COFEN).

[...] *treinamentos sobre o tema, fluxos. Isso vai levar o profissional a pensar em economia ao gerir o cuidado. Ajudará também a termos uma assistência qualificada e redução de custos.* (Enf.1)

*Uma equipe motivada facilita muito a interação do cuidado com a economia; a influência é grande*. (Enf.2)

[...] *a quantidade de tempo ideal que eu tenho disponível no meu plantão para poder orientar o meu paciente irá influenciar de forma positiva.* (Enf.5).

*O conhecimento do profissional para a execução da sua prática profissional é, sem dúvida, uma condição que influencia positivamente.* (Enf. 11)

*O conhecimento do profissional sobre o assunto*. (Enf.13; Enf.18)

*A qualidade dos materiais que nos é oferecido para atuarmos facilita essa interação* [...] *atitudes dos governantes ou órgãos que nos regem também.* (Enf.14)

*Eu acho que algo que facilita esse processo de eficiência e efetividade no trabalho da enfermagem está muito ligado à valorização profissional* [...] *o profissional valorizado trabalha melhor.* (Enf. 18)

Em outra perspectiva, a subcategoria “Condições limitadoras do processo interativo entre economia da saúde e assistência de enfermagem ao paciente hospitalizado” revelou existir condições que limitam/dificultam a interação entre esses dois polos.

Tratando-se das condições limitadoras, os resultados sinalizaram as situações institucionais que não geram motivação ao profissional de enfermagem. Para os participantes da pesquisa, a motivação constrói atitudes profissionais de impacto na ES. Nesse ínterim, foi destacada também a importância das relações interpessoais no trabalho que, entre outros fatores, mencionam os enfermeiros, sendo influenciadas pela disposição de verbas destinadas às instituições de saúde; neste caso, no contexto hospitalar.

Para os enfermeiros, as relações de trabalho podem ser afetadas pela falta/escassez de materiais para que o cuidado seja desenvolvido com qualidade. Além disso, essa escassez implica a sobrecarga da equipe, bem como a insatisfação para o trabalho.

[...] *é necessário falarmos sobre motivação. Se estamos em um local onde não existe bom relacionamento, gera desgaste físico e/ou emocional, impacta muito na relação existente entre o gerenciamento do cuidado e economia da saúde.* (Enf.2)

*Eu acho que é uma cultura que tem que ser mudada nos hospitais públicos, a de existir reservas diminuídas. Isso influencia negativamente a relação da economia e o cuidado que oferecemos ao paciente.* (Enf.7)

[...] *quando faltam materiais, dificulta muito*. (Enf.14)

[...] *a sobrecarga, a insatisfação da equipe de enfermagem, tudo isso influencia no nosso desempenho e no que iremos fazer. E quem precisa dobrar não atuará de forma insatisfeita, porque as pessoas têm outros compromissos, além de trabalhar aqui.* (Enf.15)

Além das situações supramencionadas, há um agravante que se apresenta como barreira à interação entre ES e trabalho da enfermagem no hospital. Esse fator está relacionado à fragilidade do conhecimento da enfermagem sobre ES e, também, ao suposto desconhecimento do sistema de saúde em relação às conexões entre enfermagem e ES.

[...] *digo certamente que a enfermagem, de modo geral, não conhece sobre economia da saúde. E não conhecem por não terem sido levados a pensar sobre isso. A estrutura do sistema de saúde não vê essa relação entre o gerenciamento do cuidado e economia da saúde, então nós não veremos.* (Enf.14)

## DISCUSSÃO

A dimensão simbólica dos enfermeiros sobre ES está sustentada no entendimento de que, para sua funcionalidade, há interdependência entre as esferas microeconômicas/microcultura e macroeconômicas/macrocultura^(1,18)^, inseridas desde nas condições que afetam diretamente o cuidado ao paciente até nas dimensões mais amplas e de conjunturas governamentais, por exemplo. Desse modo, o trabalho da enfermagem não está distanciado da preocupação crescente com os fatores econômicos que condicionam tanto a prestação de serviço de saúde, os próprios indicadores de saúde da população^(8,19)^. Ambas dimensões são retroalimentas entre si.

Em consonância com os resultados deste estudo, principalmente quando, para os enfermeiros, a ES se apresenta de forma multifacetada desde a redução do consumo de recursos materiais em saúde até uma prática sustentável, há evidências de que os gastos com internação e tratamento controlam os gastos com consumo de saúde com repercussões crescentes, devido ao elevado número de doenças crônicas, ao processo de envelhecimento populacional e à alta densidade tecnológica existente nas instituições hospitalares^(20)^.

Por outra perspectiva, a construção de diálogos nos espaços organizacionais para o consumo consciente de recursos, a fim de evitar desperdícios e controlar os custos, significada pelos participantes deste estudo como realidade importante, estabelece nexos com o entendimento de que o hospital é, também, contexto propício para as interações humanas afetas ao processo de conhecimento e construção de valores que direcionam a práxis sustentável no cuidado à pessoa^(20,21)^. Nesse sentido, tem-se que as ações desenvolvidas em prol do gerenciamento econômico podem estar enraizadas nos significados que os profissionais construíram e valorizam sobre as conexões entre o que desempenham e o impacto que exercem na economia^(18,21)^.

Ademais, à medida que os enfermeiros se reconhecem como força motriz para o estabelecimento e a manutenção de processos de trabalho capazes de promover a sustentabilidade no setor da saúde, mediante uso racional de recursos materiais, por exemplo, tem-se que os dados corroboram indicativos de visão crítica da profissão em relação à própria importância para a ES a partir da natureza numérica^(10,22)^, bem como da natureza ontológica para o saber-fazer da enfermagem nas práticas de cuidado que envolvem a interdependência de ações gerenciais e assistenciais típicas do gerenciamento do cuidado de enfermagem^(8,9)^. Nesse sentido, a pesquisa revelou que o gerenciamento do cuidado de enfermagem é elo, em conjuntura de microcultura, da aproximação das políticas macroeconômicas e microeconômicas no âmbito hospitalar para a indissociabilidade economia e saúde a partir do trabalho da enfermagem.

Entre as premissas do Interacionismo Simbólico, está o entendimento de que os significados sobre dado objeto são construídos socialmente a partir das interações entre as pessoas, porém tais interações ocorrem em perspectivas contextuais onde emerge e se desenvolve o objeto significado^(14)^. Nessa lógica, tratando-se dos significados que os enfermeiros desvelam sobre a ES e suas interfaces com o trabalho da enfermagem, faz-se necessário corroborar o pensamento de que é fundamental o desenvolvimento da cultura econômica nas instituições hospitalares. Depreende-se desse processo ações como cultura organizacional, processo formativo dos profissionais, ainda que em âmbito da educação permanente, fluxos e rotinas que favoreçam as tendências da cultura organizacional instituída ou prospectada, bem como ações que favoreçam *feedback* aos significados dos profissionais sobre as conexões entre seus trabalhos e ES^(9,22,23)^.

Apesar do exposto, não é incomum haver inconsistências que interferem no gerenciamento do cuidado a partir das implicações negativas decorrentes da gestão econômica ineficiente e que também modificam o campo dos significados sobre as conexões entre essas duas dimensões, de modo a afetar o próprio processo de trabalho quando as fragilidades da ES resultam, por exemplo, em dificuldades para a tomada de decisão dos profissionais com base nas melhores evidências^(9,23-26)^, sobretudo quando há escassez de recursos tecnológicos e de recursos humanos qualificados.

As condições que podem combater os obstáculos para as conexões entre ES e trabalho da enfermagem hospitalar levam em consideração a dinâmica do próprio contexto organizacional como realidade capaz de reordenar significados já instituídos no decurso cultural de dada realidade, quando, por ação intencional, projeta condições para novos olhares sobre a mesma realidade. Dessa conjuntura, depreendem-se possibilidades para um novo processo interpretativo dos sujeitos em face de novos significados para o mesmo objeto ou conjuntura^(14,26)^. Nesta pesquisa, ações como treinamentos promovidos pelo hospital relacionados à ES e fluxos que direcionem as atitudes profissionais a um cuidado sustentável e de qualidade parecem corroborar possibilidades simbólicas para projetar e/ou fortalecer significados sobre ES e enfermagem.

Cabe, desse modo, destacar que a posição epistemológica do Interacionismo Simbólico rejeita a ideia de que o mundo social pode ser representado em termos de relações determinísticas, e caminha em favor de uma visão de que o conhecimento, o entendimento e as explicações das relações sociais levam em conta a ordem social, e é elaborada pelos seres humanos, pelo modo que são significativas para eles^(14)^. Assim, posicionar-se na construção de estratégias de motivação e valorização profissional inclinada para ações que impulsionem cuidados pensados em perspectiva custo-efetividade é condição capaz de impulsionar a gestão da economia em saúde de forma eficiente. Ademais, em perspectiva macroeconômica, estão atitudes governamentais^(27,28)^, bem como atitudes dos órgãos/entidades vinculados à profissão, com destaque para o COFEN. Por essa razão, admite-se que estudos sobre o tema em tela são necessários, a fim de fornecer aos tomadores de decisão informações importantes sobre o uso efetivo dos recursos disponíveis para potencializar os benefícios da promoção da saúde^(27)^, considerando a realidade contextual e simbólica dos envolvidos nos cuidados em saúde, nos diferentes contextos onde são desenvolvidos.

### Limitações do estudo

Apesar de a pesquisa apresentar os significados dos profissionais que possuem, sob força deontológica, o papel de coordenar a equipe de enfermagem, os significados desvelados pelos demais membros da equipe de enfermagem poderiam agregar valor aos dados, de modo a sinalizar ou não outros fatores condicionantes às conexões entre ES e enfermagem. Este entendimento assume implicação contextual/nacional, pois considera a realidade brasileira, na qual a enfermagem é composta por enfermeiros, técnicos e auxiliares de enfermagem. Soma-se ao exposto o fato de os demais membros da equipe de enfermagem consumirem, em expressiva projeção, recursos materiais para a realização de cuidados de enfermagem. A realidade de hospitais privados, em virtude da cultura organizacional, pode sinalizar dados diferentes em relação ao que fora apresentado no estudo em tela.

### Contribuições para as áreas da enfermagem, saúde ou políticas públicas

Os significados desvelados pelos enfermeiros, sobre ES, a partir da realidade hospitalar, conferem possibilidades para compreendermos como esses profissionais se percebem diante do trabalho que realizam e que é afetado pela ES. Ao mesmo tempo, demonstram como a enfermagem impacta, ainda que indiretamente, a ES a partir da natureza ontológica e numérica que apresentam nas instituições hospitalares no cuidado ao paciente.

As interações simbólicas entre ES e enfermagem hospitalar sinalizam, a partir dos significados desvelados pelos enfermeiros, as condições que influenciam a enfermagem e ES, desde fluxos organizacionais, processos de conhecimento desses profissionais para a sustentabilidade de cuidado e da saúde, até medidas de valorização institucionais e governamentais capazes de contemplar a enfermagem a partir da importância que exercem para a ES.

## CONSIDERAÇÕES FINAIS

É no âmbito da micropolítica do trabalho que os enfermeiros, inicialmente, compreendem as condições intervenientes relacionadas às conexões entre ES e o trabalho que exercem no hospital. Todavia, esses profissionais também atribuem significados direcionados pela macropolítica quando reconhecem a ES como elemento político que é regido por tomadores de decisão que nem sempre alcançam as demandas da enfermagem, mas que afetam diretamente o gerenciamento de recursos materiais e humanos da saúde, notadamente no âmbito hospitalar.

Os enfermeiros se reconhecem como força de trabalho estratégica para impulsionar a ES, no contexto hospitalar, a partir das posições que assumem nesses cenários, seja em decorrência de suas permanências ininterruptas ou do quantitativo de profissionais, mas, principalmente, pela natureza do trabalho que realizam a partir do consumo e gerenciamento de recursos para a assistência ao paciente hospitalizado. Ademais, entre as condições intervenientes afetas aos significados dos enfermeiros sobre ES e o trabalho da enfermagem, estão ações que podem ser estratégicas, como educação permanente relacionada à ES, incentivos e motivação do trabalhador para melhor gerenciar recursos, lideranças e políticas de valorização da enfermagem.
